# An Introduction to the Supplemental Issue on Why Does Health in the US Continue to Lag Behind

**DOI:** 10.1093/geronb/gbac050

**Published:** 2022-05-27

**Authors:** Neil K Mehta, Mikko Myrskylä

**Affiliations:** Department of Epidemiology, School of Public and Population Health, The University of Texas Medical Branch, Galveston, Texas, USA; Max Planck Institute for Demographic Research, Rostock, Germany; Center for Social Data Science and Population Research Unit, University of Helsinki, Helsinki, Finland

Health and mortality in the United States rank poorly by international standards, despite the nation’s robust economic and technological standing. In 2010, life expectancy at birth in the United States, at 78.8 years, was 1.0 year lower than the average of 27 European Union countries (Eurostat, 2021; University of California, Berkeley and Max Planck Institute for Demographic Research, 2015). Between 2010 and 2019, the U.S. shortfall in life expectancy doubled to 2.1 years—largely a product of U.S. life expectancy growth stalling. Between 2014 and 2017 U.S. life expectancy experienced three consecutive year-over-year declines, unique among high-income countries. The coronavirus disease 2019 (COVID-19) pandemic has only served to exacerbate these preexisting shortfalls (Woolf et al., 2021). The U.S. lag is evident for both women and men and it is not attributable to the racial/ethnic diversity of the U.S. population. Even the most affluent U.S. states—those characterized by dynamic gig economies with many highly skilled workers—exhibit outcomes that are on par or lag national averages of other high-income countries (Eurostat, 2021; Montez et al., 2020). Chronic disease and disability levels are also generally higher in the United States compared to many other peer countries (Choi et al., 2020).

What are the main factors driving the U.S. shortfall in health and mortality? In 2011, and then again in 2013, the National Research Council (NRC) of the National Academy of Sciences published reports on this topic (Institute of Medicine and National Research Council, 2013; National Research Council, 2011). These reports investigated a wide range of causes including behavioral factors (cigarette smoking, caloric imbalances, violence, drug use), birth outcomes, economic and social factors, and health care system performance. The NRC volumes identified several explanatory factors including cigarette smoking, obesity, infant outcomes, violence, and drug-related mortality. These factors are themselves embedded within larger social and economic contexts, which the reports acknowledged as important contributors, but whose effects are harder to quantify compared to more proximal causes. The reports also showed that mortality under age 50 years was a larger contributor to the U.S. shortfall in life expectancy than mortality above age 50 years.

Since the NRC reports, a rich body of literature spanning demography, epidemiology, and health services has continued to investigate the U.S. shortfall. The factors that explain the U.S. shortfall may change over time and the set of causes that explain a disparity at any one point in time will often differ from the set of causes that explain changes in that disparity over time. Much of the work on this topic has also taken a period perspective—correlating factors at a point in time—while cohort-patterned forces may be critical. Thus, continued efforts at understanding the U.S. shortfall have been and continue to be well warranted.

In this Supplemental Issue, a multidisciplinary group of researchers provides an updated and wide-ranging treatment of the issue. The articles in this issue encompass studies of mortality, disability, and chronic disease. As a set, they focus on various stages of life, addressing both proximal causes as well as those related to social and economic conditions, and consider the impact of the COVID-19 pandemic. The role of cardiometabolic health features prominently across the studies.

Ho (2022) demonstrates the worsening ranking of U.S. mortality among 17 other high-income countries over time. The paper shows that between 2008 and 2018 the United States has lost ground at nearly every age with sizeable losses occurring in young adulthood. The relative advantages that the United States had at older ages also appear to be eroding. Against the backdrop of the deteriorating U.S. ranking, Masters et al. (2022) highlight the disproportionately large impact the COVID-19 pandemic had on U.S. mortality relative to peer countries. They document a 2.33 (male) and 1.69 (female) year decline in U.S. life expectancy at birth between 2019 and 2020, whereas peer countries experienced declines that averaged 0.67 (male) and 0.50 (female) years. The paper also identified that the United States experienced far greater mortality deterioration at ages 15–64 years than peer countries.

Murphy and Grundy (2022) provide an in-depth analysis of the slowdown in mortality improvements since 2000 across high-income countries and a detailed cause-specific trend analysis for the United States and United Kingdom. Among a comprehensive set of death causes analyzed, the authors find that cardiovascular disease (CVD) mortality was the major reason for the slowdown of improvements after 2010 in both countries. They discuss the complexities involved in identifying the societal causes of the slowdown in CVD mortality improvements. Acosta et al. (2022) start with the premise that stalls in CVD mortality improvements have been a principal cause of the deteriorating U.S. ranking in life expectancy. They provide a detailed analysis of CVD mortality trends in the United States and peer countries and find that the exceptionally strong stall in the United States is a function of adverse trends in multiple CVD subclassifications: ischemic heart disease, stroke, and a residual category. They provide suggestive evidence that trends in obesity and alcohol abuse are important contributors to the exceptional stall in the United States.

The above papers focus on national averages. Barbieri (2022) shifts the lens to subnational disparities. The author tracks life expectancy across deciles of U.S. counties based on socioeconomic status (SES) from 1982 to 2019. The paper clearly documents growing disparities by county-level SES. Strikingly, the study finds that for women in 2019 the average life expectancy in all 10 U.S. deciles was lower than the overall average of 20 comparison countries. For American men, only those in the top decile had a higher life expectancy than the overall average of men in comparison countries. The paper concludes that internal inequalities in U.S. mortality only partly explain the growing disadvantage in life expectancy relative to peer countries.

Two papers compare cardiometabolic health in the United States and England. Pongiglione et al. (2022) document that older adults in the United States have a much higher prevalence of diabetes, low high density lipoprotein cholesterol, and high inflammation marker levels compared to their English counterparts. While U.S. adults are also more likely to be obese, the authors provide evidence that obesity differences explain only a small fraction of the differences in the other biological risk factors. Martinson et al. (2022) analyze trends in cardiovascular risk factors among younger cohorts, comparing the Millennials (born 1981–1996) to the Generation Xers (1965–1980). The authors find that within both countries Millennials had higher obesity and prediabetes/diabetes compared to Gen Xers; however, the growth in these risk factors over generations was much more pronounced in the United States compared to in England. Levels of smoking and high cholesterol declined over the generations in both countries. The paper’s findings suggest that generational changes in obesity, income, marital status, and alcohol consumption do not explain the generational differences in either country.

Choi et al. (2022) and Daza and Palloni (2022) focus on the role of income in patterning health. Choi et al. (2022) compare trends in disability from 2002 to 2016 by income quintile in the United States and England among adults aged 55 and older. Among Americans aged 55–74 years there was no appreciable change in disability levels. Both countries experienced declines in prevalence among those over age 75 years. With respect to disparities by income, both countries experienced a widening gap in disability between low- and high-income adults driven largely by improvements in disability levels among upper-income individuals. Overall, improvements were more widespread in England compared to the United States. Daza and Palloni (2022) ask whether contextual income mobility—an aggregate measure of generational income change—to which one is exposed to in early life shapes later-life behaviors and health. Based on a careful design of county-level data aimed at mitigating confounding factors, the authors do not find strong evidence of the role of early-life income–mobility regime. Associations with cigarette smoking were demonstrated. The authors provide a rich discussion on the complexities of the causal pathways under study underscoring the importance of incorporating data from multiple periods in the life course.

This Supplemental Issue was sponsored by the TRENDS research network on old-age disability and funded by the Michigan Center on the Demography of Aging at the University of Michigan. While we are excited to see the Issue come to fruition, we are very saddened by the passing of Professor Robert F. Schoeni in October 2021 from complications of amyotrophic lateral sclerosis. Bob, as he was known to his colleagues and friends, led the TRENDS research network for 16 years. He was a distinguished scholar having made an impact on multiple social science fields including economics, sociology, and demography. Importantly, Bob was a true and early believer in interdisciplinary collaboration. His vision for TRENDS was that it includes researchers from many fields and provides opportunities for students and early-career researchers. He fulfilled that vision in many ways. Many of us, including the authors of this piece, benefitted immensely from his commitment to building interdisciplinary social science research capacity. Bob was instrumental in developing this Supplemental Issue and worked hard on its production right up until the days prior to his passing. In this Issue, Vicki Freedman and Linda Martin (2022) provide a fitting tribute to Bob’s career and legacy.

The results presented in this Issue provide a detailed account of the myriad ways health in the United States continues to lag its peers, including specifics on age groups, death causes, risk factors, and generational patterns. While details of the lag continue to be documented, the solutions continue to be evasive. Violence and drug overdoses, both important contributors to the lag, have both proximal (e.g., availability of firearms, prescribing regulations) and structural (e.g., economic, social) features, which likely interact with each other in complex ways. While CVD and its risk factors explain some of the lag and may be prominent in recent trends, appropriate interventions are challenging and require multilevel social and health care interventions. Such comprehensive approaches seem daunting to implement in current policy and political contexts. The United States not only ranks poorly in terms of health but also in terms of its investments in social welfare and levels of income inequality. Such a correlation is likely not spurious. Current social and health policies have an important role to play, but scientists are just beginning to tackle the complexities involved in identifying the effects of policies on aggregate measures of population health (Beckfield & Bambra, 2016; Montez et al., 2020). This direction is encouraging and further advances in understanding the root causes of U.S. health in comparison to peer nations might come from analysis of systemic, social, and political processes relevant for health.
